# Gingival crevicular fluid as a functionalized point of care for the early detection of dementia or the asymptomatic phase of neurodegeneration

**DOI:** 10.3389/fdmed.2025.1738635

**Published:** 2026-01-15

**Authors:** Ylenia Leanza, Antonio Belmonte, Alessandro Polizzi, Daniela Galimberti, Gianluca Martino Tartaglia, Gaetano Isola

**Affiliations:** 1 Department of General Surgery and Surgical-Medical Specialties, School of Dentistry, University of Catania, Catania, Italy; 2 International Research Center on Periodontal and Systemic Health “PerioHealth”, University of Catania, Catania, Italy; 3 Department of Biomedical, Surgical and Dental Sciences, University of Milan, Milan, Italy; 4 Fondazione IRCCS Ca’ Granda, Ospedale Maggiore Policlinico, Milan, Italy

**Keywords:** biomarkers, gingival crevicular fluid, neurodegenerative diseases, oralmicrobiome, periodontitis

## Abstract

Growing evidence links chronic systemic inflammation, particularly from periodontitis, to neurodegenerative processes, which have been reported to share common pathways. Early detection of neurodegenerative diseases such as Alzheimer's disease is crucial, given that underlying neuropathological processes evolve silently for decades before diagnosis. The gingival crevicular fluid (GCF), a serum-derived exudate from the gingival sulcus, mirrors both local periodontal inflammation and systemic conditions. Its molecular composition—rich in cytokines, enzymes, oxidative stress markers, and microbial metabolites—makes it a potential source of biomarkers reflecting neuroinflammatory pathways. This review discusses the biological rationale and emerging evidence supporting the use of GCF as a functionalized biofluid for early detection of dementia or asymptomatic neurodegeneration. By integrating advances in biosensing and lab-on-a-chip technologies, GCF analysis could become a minimally invasive, point-of-care approach to identify individuals at risk of neurodegenerative diseases. Exploring this oral–brain connection may open new perspectives in preventive medicine and personalized diagnostics.

## 1 Introduction

In recent years, increasing evidence has highlighted a potential link between chronic oral infections and neurodegenerative diseases. In this regard, it has been reported that periodontitis, a multifactorial inflammatory disease of the periodontal apparatus of the oral cavity, is strictly linked with a plethora of systemic diseases such as neurodegenerative disorders (1). Among the bacteria associated with periodontitis, *Porphyromonas gingivalis*, a keystone pathogen involved in the onset and progression of periodontal disease (1, 2), has received particular attention for its potential role in neurodegeneration (3). Several studies have detected the presence of *P. gingivalis* in the brain tissue of individuals who died with Alzheimer's disease (AD) or other causes, suggesting a possible pathogenic relationship between periodontal infection and neurodegeneration (4, 5). It has therefore been proposed that periodontitis could represent a risk factor for AD and that the invasion of *P. gingivalis* into the brain may trigger neuroinflammatory and neurotoxic effects, contributing to cognitive decline (6).

Periodontitis is a chronic inflammatory disease of the tooth-supporting tissues, characterized by a dysbiotic subgingival biofilm dominated by *P. gingivalis* (7). The bacterium and its virulence factors can breach the gingival epithelium and enter the bloodstream, triggering a systemic immune response marked by elevated levels of IL-6, TNF-α, and CRP. This persistent, low-grade inflammatory state is increasingly recognized as a biological link between peripheral infection and neurodegeneration (8, 9).

Within this context, the gingival crevicular fluid (GCF) has emerged as a promising biological matrix for identifying biomarkers that connect periodontal and neurodegenerative conditions (10). GCF is a serum-derived exudate that reflects both the local periodontal environment and systemic inflammatory changes, effectively acting as a localized liquid biopsy (11).

Chronic inflammation induced by *P. gingivalis* may activate microglia and astrocytes, promoting oxidative stress and the overproduction of *β*-amyloid (A*β*), a key neuropathological marker of AD (10, 12). This supports the hypothesis that oral pathogens can exacerbate amyloidogenesis and neuroinflammation.

Moreover, apolipoprotein E (ApoE), a crucial regulator of A*β* clearance and major genetic risk factor for AD, may be influenced by peripheral inflammation (13). Recent studies have found altered ApoE levels and higher *P. gingivalis* load in the GCF of AD patients compared to healthy controls, suggesting that this fluid could reflect both periodontal and neurodegenerative changes (10, 14, 15).

Altogether, these findings indicate that GCF could serve as a non-invasive, functionalized diagnostic fluid for the early detection of inflammatory and neurodegenerative biomarkers, offering an innovative *point-of-care* approach that bridges oral and brain health.

Therefore, this review aims to explore the biological rationale and current evidence supporting the gingival crevicular fluid as a potential diagnostic biofluid for the early detection of dementia and asymptomatic neurodegenerative changes.

## 2 Material and methods

In September 2025, a comprehensive narrative literature search was conducted without time restrictions across major electronic databases, including PubMed, Scopus, Web of Science, and Google Scholar. The search strategy combined Medical Subject Headings (MeSH) terms and free-text keywords related to periodontal disease, gingival crevicular fluid, oral microbiota, neuroinflammation, and neurodegenerative disorders. Key search terms included “gingival crevicular fluid,” “periodontitis,” “oral microbiome,” “Porphyromonas gingivalis,” “Alzheimer's disease,” “Parkinson's disease,” “neurodegeneration,” and “biomarkers,” used alone or in combination.

This narrative review was developed by selecting articles according to the following inclusion criteria: (1) articles written in English; (2) experimental and clinical studies investigating the relationship between periodontal disease, oral microbiota, and neurodegenerative disorders, with particular attention to Alzheimer's disease and Parkinson's disease; (3) studies evaluating gingival crevicular fluid and/or saliva as potential sources of inflammatory, microbial, metabolic, or neurodegeneration-related biomarkers; (4) study designs including *in vivo* and *in vitro* studies, prospective and retrospective studies, cross-sectional studies, narrative reviews, systematic reviews, and meta-analyses. Articles were excluded if they met one or more of the following criteria: (1) not available in English; (2) not relevant to the interaction between periodontal inflammation, oral microbiota, and neurodegenerative processes; (3) opinion papers, editorials, or conference abstracts without accessible full text.

## 3 GCF as a biological window

GCF is a physiological exudate that accumulates in the gingival sulcus and periodontal pockets, originating from the microcirculation of the gingival connective tissue.

Under physiological conditions, GCF flow is minimal; however, it increases significantly during inflammation due to enhanced vascular permeability and the migration of immune cells. This fluid contains a complex mixture of proteins, cytokines, enzymes, antibodies, metabolites, lipids, and bacterial components, reflecting the close interaction between host immune mechanisms and subgingival microbiota (11). Among its major constituents, pro-inflammatory mediators such as IL-1β, IL-6, and TNF-α, along with matrix metalloproteinases (MMP-8 and MMP-9), play a key role in connective tissue degradation and alveolar bone resorption (16, 17). Because of its composition and responsiveness to inflammatory changes, GCF represents a valuable diagnostic medium that mirrors both local periodontal inflammation and systemic immune alterations. Moreover, it can be collected non-invasively offering a repeatable and convenient sampling method, though its composition may vary depending on the degree of inflammation and the sampling site (11).

Recent research has expanded the diagnostic relevance of GCF beyond periodontal disease, suggesting that it may also provide insights into systemic conditions such as neurodegenerative disorders.

Elevated levels of IL-1β, IL-6, and TNF-α detected in GCF have been associated not only with periodontal tissue destruction but also with chronic low-grade inflammation implicated in neurodegeneration (10).

**Figure 1:**
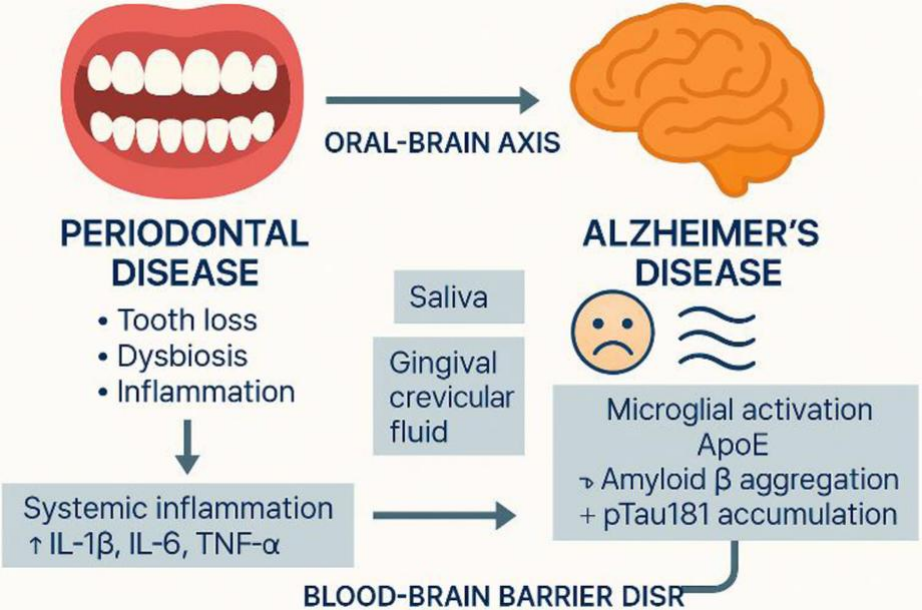
A possible link between periodontal disease and neurodegeneration through the oral–brain axis.

In patients with Parkinson's disease, studies have demonstrated increased *P. gingivalis* load, higher oxidative stress markers, and elevated concentrations of pro-inflammatory cytokines and complement proteins in GCF, supporting a microbial and inflammatory contribution to neuroinflammatory pathways (18). Furthermore, molecules typically related to AD pathology, including *β*-amyloid (A*β*) and apolipoprotein E (ApoE), have been identified in GCF (19). Altered ApoE levels and the presence of *P. gingivalis* virulence factors in AD patients suggest that this fluid could reflect early inflammatory and metabolic changes that parallel neurodegenerative processes (Figure 1).

Compared to other biological fluids, such as cerebrospinal fluid (CSF) or blood, saliva and GCF offer a valid, noninvasive platform for identifying biomarkers associated with AD (20). CSF remains the gold standard for detecting biomarkers such as A*β* and tau proteins, but requires an invasive lumbar puncture (21). Blood testing, although less invasive, often lacks specificity due to peripheral interference (22). Saliva and GCF offer accessibility, are noninvasive, and are repeatable, reflecting both periodontal conditions and systemic inflammatory states. Saliva, unlike GCF, suffers from variability in flow rate and contamination (20).

Periodontal inflammation driven by *Porphyromonas gingivalis* triggers systemic release of IL-1β, IL-6, and TNF-α, promoting blood–brain barrier disruption, microglial activation, and the accumulation of *β*-amyloid, pTau181, and altered ApoE expression, ultimately contributing to cognitive decline.

Bidirectional arrows highlight the oral–brain interplay and the potential of gingival crevicular fluid (GCF) as a minimally invasive source of biomarkers—such as *P. gingivalis* antigens, cytokines, and ApoE—reflecting neuroinflammatory processes (20).

These characteristics make GCF a promising point-of-care biofluid for the early detection of inflammatory and neurodegenerative biomarkers, offering an innovative and accessible bridge between oral and brain health.

## 4 Clinical evidence and diagnostic potential

GCF, as a modified serum transudate that reflects both periodontal and systemic conditions, is emerging as a promising, non-invasive source for the identification of biomarkers useful in the early diagnosis of neurodegenerative diseases, particularly AD and Parkinson's disease (DP) (23).

CSF biomarkers remain the reference standard for AD, but require lumbar puncture (21), while blood tests are less invasive yet prone to peripheral interference (22). In this context, GCF is non-invasive and repeatable, and already shows quantifiable neurodegeneration-related signals: A*β* peptides (23), ApoE-*ε*4 with good discrimination across cognitive states (AUC 0.849) (10), and multi-omic GCF metabolic signatures with AUC > 0.98 in aMCI/AD stratification (24). Salivary lactoferrin provides an additional benchmark (AUC up to 0.97; sensitivity >87%, specificity >91%) (25). Overall, these performance metrics support GCF as a promising screening biofluid, although standardization and external validation remain necessary.

Periodontitis (PD) is recognized as a chronic, low-grade infectious and inflammatory disease characterized by dysbiosis of the subgingival microbiota, which has been linked to the onset or progression of a wide range of chronic noncommunicable diseases (10). The hypothesis that periodontitis may be a modifiable risk factor for cognitive decline is supported by growing evidence (26).

Case-control studies have observed a statistically significant association between clinical attachment loss (CAL) and cognitive impairment [including Mild Cognitive Impairment (MCI), and dementia], even after adjusting for confounding factors such as age and sex. Chronic periodontal damage is believed to be plausibly associated with neurodegeneration through mechanisms involving inflammation and vascular damage, as periodontal-derived inflammatory mediators (such as IL-1β, IL-6, TNF-α) can enter the systemic circulation and contribute to cerebral neuroinflammation (27).

Specifically, AD patients have been found to have more severe periodontitis (in one study, 80% of AD patients had stage III-IV periodontitis) compared to healthy subjects. In these AD patients, levels of proinflammatory mediators (such as IL-1β, IL-9, and IL-22) and the bacterial load of P. gingivalis in the GCF were significantly increased. *P. gingivalis* load and proinflammatory mediators have been shown to negatively correlate with MoCA cognitive test scores (10). The presence of *P. gingivalis* is particularly relevant, as its DNA and virulence factors have been detected in the brain tissue of AD patients, suggesting a potential causal role in the etiopathogenesis of the disease through the induction of neuroinflammation and the accumulation of amyloid *β* (A*β*) (25)**.**

In the context of DP, although patients in early or mid-stage could maintain good oral hygiene, patients with PD and periodontitis still showed a significantly increased periodontal inflammatory burden, evidenced by increased bleeding on probing (BOP) and elevated levels of pro-inflammatory cytokines in the GCF (such as IL-1β and IL-1RA) compared to controls (28).

GCF is emerging as an excellent point-of-care diagnostic tool in the early, asymptomatic, or prodromal stages of neurodegeneration. In recent years, several multi-omic studies have comprehensively analyzed the profiles of the subgingival microbiome and the GCF metabolome in patients with AD and amnestic mild cognitive decline (aMCI), with findings of significant diagnostic and pathogenetic relevance (24).

It is now clear that microbial dysbiosis is closely associated with cognitive decline. Analyses of the subgingival and salivary microbiome have shown that certain bacterial species, including *Veillonella parvula* (*V. parvula*) and *Dialister pneumosintes*, are significantly enriched in patients with AD (29). In particular, *V. parvula*—a Gram-negative anaerobic coccus—has been correlated with lower MMSE scores and the typical clinical picture of AD, both in saliva samples and in GCF. This species, known for its pathogenic potential in intracranial infections, may contribute to neuroinflammation through microglial activation, thus exacerbating neurodegenerative processes (24).

Among the most interesting biological markers identified in GCF are:

• Apolipoprotein E (ApoE-*ε*4): the main genetic risk factor for AD, it was recently quantified in GCF for the first time. Patients with AD and periodontitis have significantly higher levels of ApoE-*ε*4 than healthy controls. Its detection in GCF showed high diagnostic accuracy, with an AUC of 84.9%, in discriminating against different cognitive states, suggesting a potential role as a noninvasive biomarker of neurodegeneration (10);
• GCF Metabolites: LC-MS/MS analysis identified 165 metabolites with differential levels among healthy subjects, patients with aMCI, and AD. Integrated multi-omics analysis (DIABLO) highlighted a strong correlation between metabolites such as galactinol, sn-glycerol 3-phosphoethanolamine, D-mannitol, and L-iditol and microbial species enriched in AD (*V. parvula, D. pneumosintes*). These metabolites showed exceptional predictive accuracy (AUC > 0.98) in distinguishing AD progression in cognitively normal subjects or those with aMCI (20);
• Salivary Lactoferrin (Lf): Although a fluid distinct from GCF, lactoferrin is an important antimicrobial peptide in saliva, part of the first line of innate immune defense. Its levels are significantly reduced in the prodromal phases and in AD dementia, effectively distinguishing these subjects from those with frontotemporal dementia (FTD) and healthy controls, with a sensitivity and specificity greater than 87% and 91%, respectively. The reduction in Lf may reflect a weakening of mucosal defenses, favoring pathogenic colonization and thus contributing to the onset of neurodegenerative processes (25);
• Neurofilament light chain (NfL): A well-established marker of axonal damage, it was recently identified in the GCF of patients with DP. NfL levels in samples taken from shallow gingival sites are significantly higher in DP patients with periodontitis than in healthy controls, suggesting early involvement of periodontal tissue in the neuroinflammatory processes associated with DP (28).

**Figure 2:**
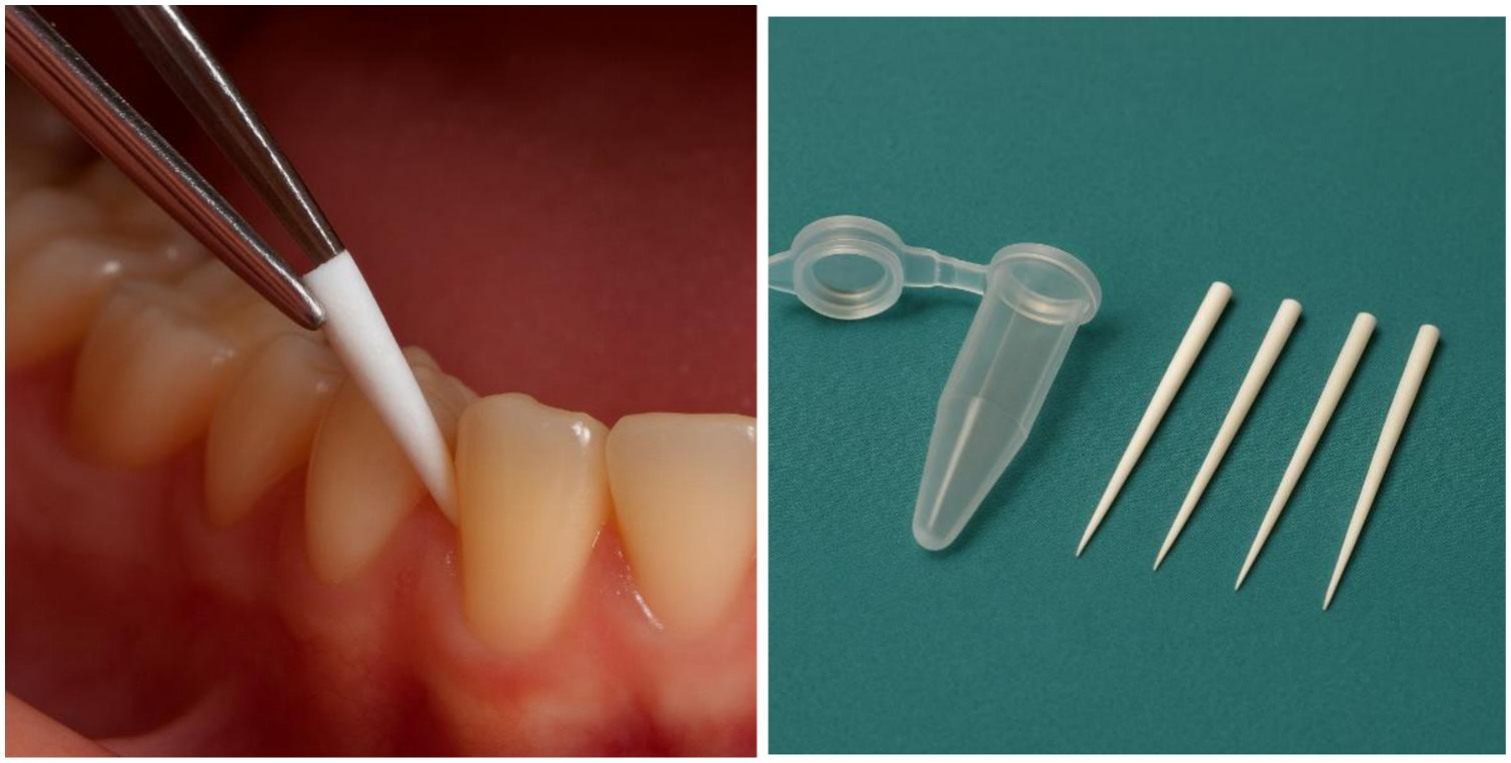
Collection of gingival crevicular fluid (GCF) using sterile paper points.

The most significant and innovative aspect of using GCF and saliva lies in their non-invasive and easily accessible nature, making them ideal tools for early screening and monitoring of neurodegenerative diseases. Unlike traditional methods such as CSF analysis or PET, which require expensive and invasive procedures, collecting GCF and saliva is simple, cost-effective, and well-tolerated by patients, paving the way for more widespread and sustainable point-of-care diagnostics (30) (Figure 2).

The sample is obtained by gently inserting sterile paper points into the gingival sulcus for a few seconds to absorb the crevicular fluid. This simple and minimally invasive technique allows for safe and reproducible collection of biological material suitable for biomarker analysis in both periodontal and systemic research contexts. Original illustration created by the author.

An additional strength lies in the integration of multi-omics approaches, which combine microbiome and metabolome analysis of GCF. This strategy allows for the simultaneous capture of local alterations (related to periodontitis), systemic changes (inflammatory processes), and molecular changes (biomarkers of neurodegeneration), thus providing a comprehensive view of the connection between oral health and brain health (20).

The discovery of specific biomarkers, such as ApoE-*ε*4 and certain discriminatory metabolites, with high diagnostic accuracy (AUC > 0.98), strengthens the clinical potential of these fluids as predictive tools for the early identification of subjects at risk of Alzheimer's disease or other neurodegenerative diseases (10).

Despite these promising prospects, there are some significant limitations that must be considered. Most studies investigating the link between periodontitis, GCF, and cognitive decline are based on cross-sectional or case-control designs, which do not allow for definitive causality or the temporal sequence of events (i.e., whether oral dysbiosis precedes or follows the onset of dementia) (24)**.**

Furthermore, there is still a lack of methodological standardization in the collection and processing of GCF and saliva. Differences in protocols—such as the use of stimulated or unstimulated collection, or different centrifugation speeds—make it difficult to compare results across laboratories and studies (30).

Many studies have small sample sizes and significant participant heterogeneity, particularly with regard to medication use, comorbidities, or periodontal status, factors that can significantly influence the observed microbial and metabolic profiles (25).

To overcome these limitations and clarify whether alterations in the microbiota and metabolome play a causal or consequential role in the progression of cognitive decline, it will be necessary to conduct large-scale longitudinal studies and cohort studies that integrate multi-omic analyses and careful standardization of experimental protocols. Only through approaches of this type will it be possible to consolidate the value of GCF and saliva as reference bioliquids in the early diagnosis and prevention of neurodegenerative diseases (20).

## 5 Point-of-care analytics technologies and perspectives

Biomarker analysis in GCF and saliva leverages several highly sensitive platforms to detect proteins and microbial profiles associated with neurodegeneration (30).

Current methods for analyzing oral biofluids include advanced techniques such as ultra-sensitive immunoassays (e.g., Simoa, used to measure T-tau in saliva) and ELISA methods to quantify proteins such as amyloid-*β* (A*β*) and lactoferrin (Lf) (29).

For complex molecular profiles, proteomic and metabolomic analysis are employed (30).

In recent studies, liquid chromatography coupled to tandem mass spectrometry (LC-MS/MS) has been used to profile hundreds of metabolites in GCF from patients with AD and aMCI (24)**.**

For microbial biomarkers, GCF and subgingival plaque are analyzed by next-generation sequencing (NGS) of the 16S rRNA gene.

Techniques such as qPCR (quantitative polymerase chain reaction) allow for the absolute quantification of key pathogens such as Porphyromonas gingivalis. Furthermore, multiplex immunoassays are essential for simultaneously quantifying a broad spectrum of inflammatory mediators (cytokines such as IL-1β, IL-6, TNF-α) present in GCF, crucial for assessing periodontal inflammatory burden (10).

The search for noninvasive and accessible biomarkers has highlighted the potential of GCF/saliva for widespread clinical use. Saliva, and by extension GCF, has been proposed as an easily collectible source for the diagnosis and risk assessment of various pathological conditions (28).

The use of an EG-ISFET (Extended Gate Ion-Sensitive Field-Effect Transistor) biosensor for noninvasive screening of AD based on salivary sugar detection has been studied. The goal of identifying markers with high diagnostic accuracy, such as GCF metabolites (AUC > 0.98), is a fundamental step towards the development of miniaturized platforms (lab-on-a-chip) capable of integrating multi-omics (microbiome and metabolome) analysis at the point-of-care (POC) level (20).

A key challenge in advancing this field is the lack of standardization in collection methods (stimulated vs. unstimulated), preprocessing (centrifugation speed), and storage of saliva/GCF samples.

The proteome composition, in fact, varies significantly between unstimulated and stimulated saliva and between secretions from different glands (30)**.**

Furthermore, obtaining adequate samples can be difficult in elderly or cognitively impaired patients, especially due to xerostomia (dry mouth) or reduced ability to cooperate. To overcome these limitations, some studies suggest the preferential use of stimulated saliva (29)**.**

The clinical prospects are promising: GCF is considered a noninvasive peripheral biofluid, anatomically closer to CSF, making it an excellent platform for detecting highly diluted analytes in the blood (24).

The POC concept, based on rapid, inexpensive, and noninvasive tests, aligns perfectly with the use of GCF for the early diagnosis of neurodegeneration (25)**.**

• Highly discriminatory biomarkers: Salivary lactoferrin (Lf) has emerged as a potential cost-effective and easily accessible screening tool, showing excellent diagnostic performance in detecting prodromal AD, distinguishing it from healthy controls and other dementias (such as frontotemporal dementia, FTD), with sensitivity and specificity exceeding 87% and 91%, respectively (AUC up to 0.97) (29);
• Microbial and metabolic markers: Oral dysbiosis, characterized by the increase in species such as *Veillonella parvula (*associated with AD in saliva and GCF) and *P. gingivalis* (associated with AD in GCF), is sensitive to cognitive changes. The detection of specific GCF metabolites (such as galactinol and D-mannitol) with high diagnostic accuracy (AUC > 0.98) to distinguish AD from aMCI/controls suggests the possibility of developing POC tests that integrate microbial and metabolic profiles (24);
• Inflammatory and risk markers: The increase in Apolipoprotein E-ɛ4 (ApoE-ɛ4) in the GCF of AD patients (with a diagnostic AUC of 84.9%) and its negative correlation with cognitive status (MoCA) make it a quantifiable risk indicator for a POC (10).

Therefore we can say that the GCF, thanks to its accessibility and the wealth of inflammatory, microbial, and metabolic markers related to brain pathology, offers an ideal platform for the development of POC devices for mass screening and early diagnosis of the pre-symptomatic phase of neurodegeneration (30).

## 6 Conclusion

Increasing evidence indicates that GCF represents a promising functionalized biofluid for the early identification of biomarkers associated with neurodegenerative processes. Its serum transudate nature reflects not only the periodontal inflammatory state but also systemic and neuroinflammatory conditions, allowing for the detection of molecular alterations that precede the onset of cognitive symptoms. Biomarkers such as apolipoprotein E-*ε*4 (ApoE-*ε*4), NfL, proinflammatory cytosines (IL-1β, IL-6, TNF-α), discriminating metabolites (galactinol, D-mannitol), and microbial components (*P. gingivalis, V. parvula*) have shown high diagnostic accuracy in distinguishing cognitively normal subjects from those with MCI and AD.

These findings support the hypothesis that oral dysbiosis and chronic inflammation represent a modifiable risk factor for neurodegeneration and that GCF may constitute a “biological bridge” between oral health and brain health.

The use of GCF as a POC opens significant prospects for the primary and secondary prevention of neurodegenerative diseases. The ability to monitor inflammatory and metabolic markers through non-invasive and repeatable sampling allows for the identification of at-risk individuals in a pre-symptomatic phase, promoting personalized and timely intervention strategies.

The integration of lab-on-a-chip technologies and multimodal biosensors could transform periodontal diagnostics into a decentralized neurobiological screening system, economically sustainable and suitable for use in routine clinical practice. In this context, collaboration between dentists, neurologists, and omics researchers becomes crucial to implement integrated prevention and surveillance pathways.

Despite promising results, further studies are needed to consolidate the value of GCF as a biomarker of neurodegeneration. Specifically:

• Longitudinal studies of large cohorts should clarify the temporal sequence between oral dysbiosis, systemic inflammation, and cognitive decline, distinguishing causal relationships from merely associative ones;
• Standardizing GCF collection, storage, and analysis procedures is essential to reduce inter-laboratory variability and ensure comparability of results;
• The integration of multi-omics approaches (metabolomics, proteomics, microbiomics) and network analysis can provide a systemic view of the involved oral and brain processes;
• The development of highly sensitive portable biosensors and AI-based point-of-care platforms will enable the translation of laboratory results into rapid and accessible clinical tools;
• Finally, exploring targeted interventions to modulate the oral microbiome (through probiotics or selective antimicrobial therapies) could open new therapeutic avenues in the prevention of neurodegeneration.

Understanding the bidirectional dialogue between the oral cavity and the brain and the use of GCF as a diagnostic biofluid represents an emerging paradigm of precision medicine. Consolidating this perspective will require an interdisciplinary approach, based on longitudinal evidence and next-generation technologies, capable of transforming dementia prevention from reactive to proactive.
